# A 9‐Year Longitudinal Study of Basilar Artery Diameter

**DOI:** 10.1161/JAHA.118.011154

**Published:** 2019-02-23

**Authors:** Mariko Takeuchi, Kaori Miwa, Makiko Tanaka, Yi Zhou, Kenichi Todo, Tsutomu Sasaki, Manabu Sakaguchi, Kazuo Kitagawa, Hideki Mochizuki

**Affiliations:** ^1^ Department of Neurology and Stroke Center Osaka University Graduate School of Medicine Suita, Osaka Japan; ^2^ Department of Cerebrovascular Medicine National Cerebral and Cardiovascular Center Suita, Osaka Japan; ^3^ Department of Stroke Medicine Hoshigaoka Medical Center Osaka Japan; ^4^ Laboratory of Pharmainformatics and Pharmacometrics Osaka University Graduate School of Pharmaceutical Sciences Suita, Osaka Japan; ^5^ Department of Neurology Osaka General Medical Center Osaka Japan; ^6^ Department of Neurology Tokyo Women's Medical University Tokyo Japan

**Keywords:** basilar artery, cardiovascular disease risk factors, cardiovascular events, cerebral small‐vessel disease, magnetic resonance imaging, Magnetic Resonance Imaging (MRI), Cerebrovascular Procedures

## Abstract

**Background:**

Dilatation of the basilar artery (BA) has been recognized as a predictor of cardiovascular events (CVEs). However, it is unclear if the longitudinal change in BA diameter (ΔBA) is associated with CVEs.

**Methods and Results:**

In a cohort of Japanese participants with vascular risk factors in an observational study, we evaluated the relationship of ΔBA to CVEs and the time course of the BA diameter. The short axis of the BA diameter was measured at the midpons level in T2‐weighted images. Brain magnetic resonance imaging measurements included cerebral small‐vessel disease, lacunars, and white matter hyperintensities. First, 493 patients were analyzed by the time‐dependent Cox proportional hazards model to evaluate the association between ΔBA and CVEs, with adjustment for age, sex, vascular risk factors, and magnetic resonance imaging parameters. Second, we assessed the longitudinal ΔBA in 164 patients who underwent long‐term follow‐up magnetic resonance imaging, by linear regression analysis. In the mean follow‐up of 8.7 years, 105 patients developed CVEs. A smaller ΔBA was independently associated with the high incidence of CVEs (hazard ratio, 0.36; 95% CI, 0.16–0.78; *P*=0.010; n=493). After a mean interval of 9.4 years, the average ΔBA was 0.41±0.46 mm (excluding patients with fetal‐type circle of Willis). Progression of BA dilatation was associated with men but inversely associated with initial BA diameter and fetal‐type circle of Willis (n=164).

**Conclusions:**

BA diameter increased over time (excluding the patients with fetal‐type circle of Willis), whereas ΔBA was inversely associated with the incidence of CVEs.

**Clinical Perspective**

**What Is New?**

- This is the first study to demonstrate longitudinal change in basilar artery (BA) diameter.
- In this study, we found that the BA diameter gradually increased over a median period of 9 years in patients with the adult or other‐type circle of Willis.
- A relationship between the change in BA diameter and cardiovascular events was assessed, and a smaller change in BA diameter was independently associated with the high incidence of cardiovascular events.

**What Are the Clinical Implications?**

- The change in BA diameter could be a potential predictor of cardiovascular events.

Intracranial arterial dolichoectasia (IADE) is defined as an increase in the length and diameter of intracranial arteries.^1^, ^2^ IADE is of increasing interest regarding the predictive value for cardiovascular events (CVEs).^3^, ^4^ Previous studies have reported associations between IADE and aging and vascular risk factors,^3^, ^5^ such as hypertension, a history of stroke or myocardial infarction, and cerebral small‐vessel disease.^6^, ^7^, ^8^ Among IADE, the basilar artery (BA) has the highest prevalence of dolichoectasia.^5^ On the other hand, mild or moderate BA dilatation is more frequent than BA dolichoectasia.^1^, ^9^ We have previously reported that the BA diameter at baseline is an independent predictor of the incidence of CVEs in a dose‐dependent manner, despite the upper limit of normal of the BA diameter.^10^ However, the time course of the BA diameter is poorly understood. Furthermore, an association between the change in BA diameter (ΔBA) and the incidence of CVEs is also unclear.

There are 2 main objectives of this study. First, we investigated whether ΔBA is related to the incidence of CVEs by combining data of consecutive magnetic resonance imaging (MRI) assessments for each individual to better define the clinical impact of the BA diameter. Second, we assessed whether the ΔBA is related to aging and vascular risk factors during follow‐up.

## Methods

The data that support the findings of this study are available from the corresponding author on reasonable request.

### Subjects

This study originates from the OSACA2 (Osaka Follow‐Up Study for Carotid Atherosclerosis, Part 2). The detailed study design has been described elsewhere.^10^, ^11^, ^12^ Briefly, subjects had ≥1 vascular risk factor, such as hypertension, diabetes mellitus, dyslipidemia, smoking history, or a history of cardiovascular disease (CVD). All participants underwent a baseline clinical assessment that included medical history, an inquiry about medications and smoking habits, a physical and a neurological examination, blood sampling, and a carotid ultrasound. In our previous study,^10^ among 1106 outpatients in the OSACA2 between January 2001 and June 2007, 493 patients who underwent brain MRI and were aged ≥50 years were included.

First, in the current study, extended follow‐up data were collected between June 2011 and June 2016 from all 493 patients of our previous study^10^ to determine the incidence of CVEs and obtain information on consecutive MRI assessments during the follow‐up period (Figure S1). Subjects visited outpatient clinic settings regularly to control risk factors to prevent CVD and for repeated medical checks that included carotid ultrasound and MRI images.^13^ Follow‐up was terminated when patients died (n=48) or withdrew for personal reasons (n=63).

Second, among them, 168 patients who underwent multiple MRI assessments for >7.5 years of follow‐up were investigated to elucidate the relevant clinical factors associated with ΔBA. Patients with inappropriate MRI sequences (n=4) were excluded. All available longitudinal data (n=164) were used for analysis (Figure S1).

This study was conducted in accordance with the Declaration of Helsinki and approved by the local ethics committee (307 and 8188). All participants provided written informed consent.

### MRI Protocol and Assessment

At baseline and follow‐up, MRI was performed with a 1.5‐T instrument (Signa; GE Healthcare, Milwaukee, WI) while the patient was supine. The image protocol included T1‐weighted, fluid‐attenuated inversion recovery sequence, and T2‐weighted images. The short axis of the BA diameter was measured at the midpons level on the axial T2‐weighted image^2^, ^10^ (repetition time/echo time, 5000/130 ms; flip angle, 20°; matrix, 256×256; field of view, 220 mm; slice thickness, 5 mm; and interslice gap, 1.5 mm). MRI was mostly performed to examine lesions in individuals with a history of stroke or suspicious neurological symptoms (eg, headache, vertigo, dizziness, numbness, syncope, or subjective memory impairment) during the follow‐up period. If patients underwent additional MRI between baseline and the last follow‐up MRI, all data were included in the analysis.

ΔBA was the difference of the BA diameter between 2 points on MRI assessments and was normalized as the annual ΔBA (ΔBA/y). White matter hyperintensities (WMHs) were defined as hyperintense signal abnormalities surrounding the ventricles and the deep white matter on fluid‐attenuated inversion recovery sequence images. The degree of WMHs was visually rated using the Scheltens scale,^14^ with slight modifications. That is, scores of 0 to 6 were given for deep WMHs (DWMHs) of the frontal, temporal, parietal, and occipital lobes (range, 0–24), and scores of 0 to 2 were given for the extent of hyperintensity along the frontal horn caps, occipital horn caps, and white matter bands along the lateral ventricles (periventricular hyperintensity [PVH]; range, 0–6). The sum of the PVH and DWMH scores was the total WMH score, which ranged from 0 (absent) to 30 (maximal severity). Lacunar infarctions were defined from 3 to 15 mm, with a hypointense lesion and hyperintense rim on fluid‐attenuated inversion recovery sequence images when located supratentorially in the subcortical white matter, thalamus, or basal ganglia, according to the corresponding hyperintensity on T2‐weighted images and hypointensity on T1‐weighted images. The type of the posterior circle of Willis was assessed using MR angiograms. We categorized the type of the posterior circle of Willis into 3 types: adult type (diameter of the P1 segment was larger than that of the same side posterior communicating artery bilaterally), fetal type (diameter of the posterior communicating artery was larger than that of the same side P1 segment bilaterally), and “other” type (other variations).^15^ All measurements were performed by 2 expert stroke neurologists (M.T., M.T.) who were blinded to patient data. An intrareader reproducibility study, based on 50 random MRI scans, was performed at the end of the study; the intraclass correlation coefficient of BA diameter measurement was 0.88.

### Clinical Factors

Blood pressure and body mass index were measured in outpatient clinics at baseline and at a follow‐up visit. Medical history included hypertension (defined as a casual systolic blood pressure of ≥140 mm Hg, a diastolic pressure of ≥90 mm Hg, or the current use of antihypertensive agents), diabetes mellitus (defined as a fasting blood glucose level of ≥126 mg/dL [≥7 mmol/L], a glycosylated hemoglobin A1c concentration of ≥6.5%, or the current use of insulin or oral hypoglycemic agents), dyslipidemia (defined as a fasting triglyceride level of ≥150 mg/dL [≥1.7 mmol/L], a low‐density lipoprotein cholesterol level of ≥140 mg/dL [≥3.6 mmol/L], a high‐density lipoprotein cholesterol level of ≤40 mg/dL [≤1.1 mmol/L], or the current use of lipid‐lowering drugs), and CVEs (obtained from the clinical records of patients at baseline and at a follow‐up visit). CVEs included coronary heart disease (myocardial infarction, angina requiring hospitalization, coronary artery bypass surgery, or coronary artery angioplasty), cerebrovascular disease (stroke [cerebral infarction, intracerebral hemorrhage, or subarachnoid hemorrhage] or transient ischemic attack, defined as sudden focal neurological deficits lasting <24 hours and not associated with cerebral infarction on a computed tomographic or MRI scan), and arteriosclerosis obliterans requiring hospitalization. Smoking history and current medication prescriptions were assessed. The estimated glomerular filtration rate was calculated using the Modification of Diet in Renal Disease formula for Japanese patients.^16^

### Evaluation of Extracranial Carotid Atherosclerosis

Duplex carotid ultrasonography was performed to evaluate the severity of extracranial carotid atherosclerosis at baseline. The mean maximum intima‐media thickness of 12 carotid segments (near and far walls of the right and left distal common carotid arteries, carotid bifurcation, and internal carotid artery) was assessed as a marker of systemic atherosclerosis.^17^ All examinations were performed by expert stroke neurologists who were blinded to patient clinical data.

### Statistical Analysis

Statistical analyses were performed using JMP 12.0 (SAS Institute Inc, Cary, NC) and R.3.4.2 (https://www.r-project.org/). Clinically significant variables were selected as follows: age, sex, vascular risk factors, and MRI parameters. Vascular risk factors included hypertension, diabetes mellitus, dyslipidemia, current smoking, and a history of CVD; MRI parameters were assigned as the BA diameter, PVH and DWMH scores, and the presence of lacunar infarctions or the type of the posterior circle of Willis. In the study of longitudinal ΔBA in 164 patients, if they underwent treatment for more than half of the follow‐up period, the patient was categorized as a patient with hypertension/dyslipidemia/diabetes mellitus during the observational period.

The time‐dependent Cox proportional hazards model was used as survival data in 493 patients. Time‐dependent covariates were variables described above, as well as the changes of all adjacent BA diameters per patient. The censored times were discovered at the onset of CVEs, at the follow‐up of MRI, or at the end point of the study. The hazard ratio (HR) was used to estimate the risk of CVEs using the Cox proportional hazards models after adjusting for age and sex and after adjusting for age, sex, and vascular risk factors, which was then further adjusted for MRI parameters. To identify to what extent the ΔBA applied to the risk of CVEs, we further adjusted for initial BA diameter in the final model. In the analysis of longitudinal ΔBA in 164 patients, linear regression analyses were performed to examine the variables associated with ΔBA/y. In the multivariate analysis, the included covariates were factors with a *P*<0.20 in the univariate analysis. For group comparisons between those with and without the incidence of CVEs, we used 2 tests for categorical variables and the Student *t* test for continuous variables. We also performed a preplanned sensitivity analysis by the type of circle of Willis. The comparison of the BA diameter on the slice of T2‐weighted images and time‐of‐flight MR angiograms was examined using intraclass correlation coefficients and Bland‐Altman plots. *P*<0.05 was considered significant.

## Results

### ΔBA (change in BA diameter) and the Incidence of CVEs: the Longitudinal Study of 493 Patients

A clinical baseline of 493 patients (mean±SD age at baseline, 68±8 years; 58% men) are shown in Table S1.^10^ The mean±SD follow‐up period was 8.7±3.8 years; MRI follow‐up occurred in 293 patients. A total of 228 patients underwent 2, 28 patients underwent 3, and 37 patients underwent ≥4 MRI scans. No significant differences in sex or vascular risk factors were observed between patients with and without follow‐up MRI (n=293 and n=200, respectively). However, the age of patients with follow‐up MRI was younger, and body mass index was higher, than that of patients without follow‐up MRI. A total of 105 patients (21.3%) developed CVEs during the follow‐up period. Of these 105 patients, 58 developed cerebrovascular events (transient ischemic attack in 7, lacunar infarction in 15, large‐artery atherosclerosis in 14, cardioembolism in 9, other or unknown infarction in 10, cerebral hemorrhage in 2, and subarachnoid hemorrhage in 1); 41 patients developed coronary events (acute myocardial infarction in 10 and revascularization therapy for ischemic heart disease in 31), and 6 patients experienced peripheral arterial events (Table S2).

**Figure 1:**
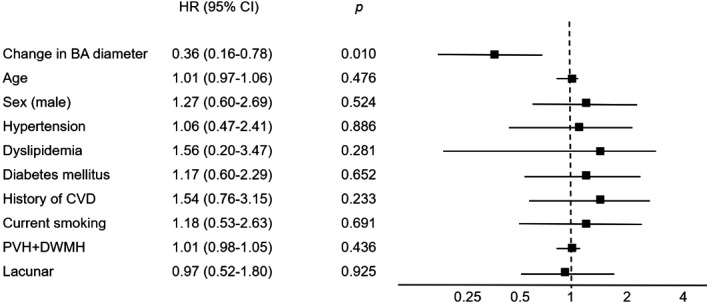
Hazard ratio (HR) used to estimate the risk of cardiovascular events among changes in basilar artery (BA) diameter, traditional vascular risk factors, and magnetic resonance imaging parameters. CVD indicates cardiovascular disease; DWMH, deep white matter hyperintensity; PVH, periventricular hyperintensity.

Cox regression with time‐dependent covariates showed that ΔBA had an inverse association with CVEs after adjustment for age and sex (HR, 0.39; 95% CI, 0.19–0.80; *P*=0.011); after adjustment for age, sex, and vascular risk factors (HR, 0.44; 95% CI, 0.21–0.90; *P*=0.024); and after further adjustment for MRI parameters (ie, lacunar infarction and total WMH score) (HR, 0.36; 95% CI, 0.16–0.78; *P*=0.010; Figure 1). After adding the data of the initial BA diameter in the final model, the association between ΔBA and CVEs disappeared. Among CVEs, coronary events were associated with ΔBA after adjustment for age and sex (HR, 0.17; 95% CI, 0.05–0.62; *P*=0.010) but cerebrovascular events were not (HR, 1.02; 95% CI, 0.31–3.32; *P*=0.970).

To prevent the effect of anatomic variation, we also assessed the association between ΔBA and CVEs in patients without the fetal‐type circle of Willis (n=473). Of these patients, a ΔBA (HR, 0.41; 95% CI, 0.16–1.08; *P*=0.072) and a history of CVD (HR, 2.21; 95% CI, 1.05–4.68; *P*=0.038) were related to the risk of total CVEs after adjusting age, sex, vascular risk factors, and MRI parameters.

### The Longitudinal Changes in BA Diameter

**Figure 2:**
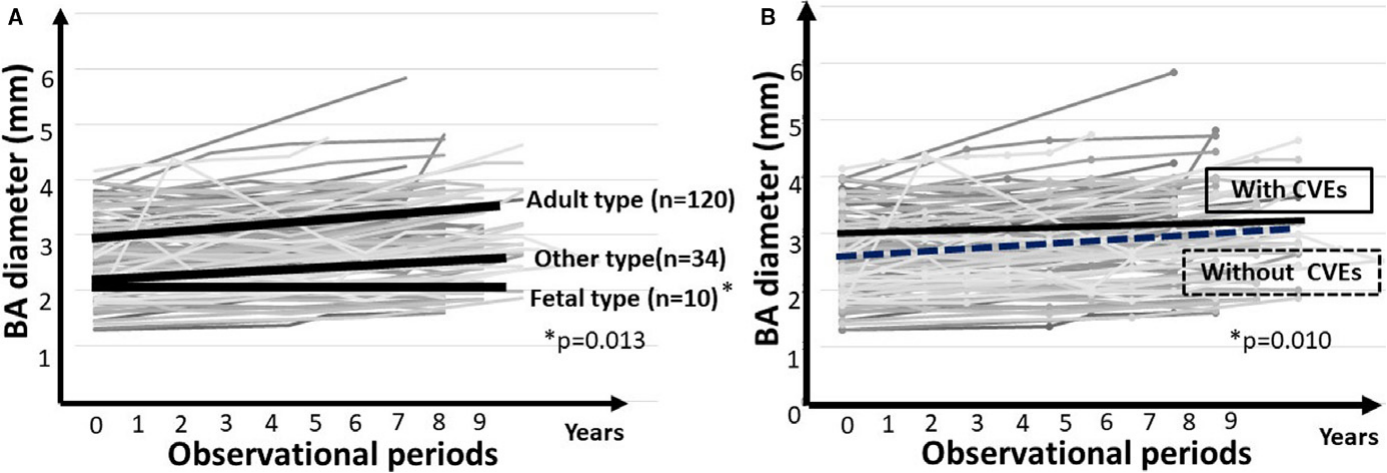
Transition of basilar artery (BA) diameter. **A**, BA diameter increased in patients with adult and other‐type circles of Willis during the follow‐up period. **B**, The change in BA diameter of patients without cardiovascular events (CVEs) is greater than that in patients with CVEs.

**Figure 3:**
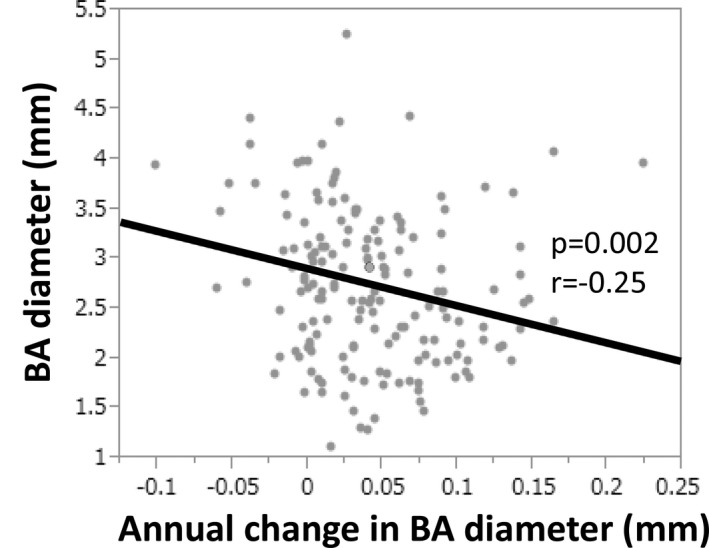
Inverse correlation between basilar artery (BA) diameter and change in BA diameter.

For the analysis of 164 patients (mean±SD age at baseline, 65±8 years; 59% men), the mean±SD MRI interval (between initial and last MRI) was 9.4±1.3 years. The characteristics are listed in Table 1. Each conventional vascular risk factor was well controlled throughout the follow‐up period. As for the type of the posterior circle of Willis, 120 patients (73%) had the adult type, 10 patients (6%) had the fetal type, and 34 patients (21%) had the other type. The average initial and last follow‐up BA diameters were 2.9±0.7/2.0±0.5/2.1±0.6 mm and 3.4±0.7/2.0±0.3/2.5±0.6 mm, respectively (Table 1). There were no significant differences in sex, history of CVD, incidence of CVEs during the follow‐up period, BA diameter, or prevalence of fetal‐type circle of Willis between participants and nonparticipants. However, the age, PVH score, and DWMH score of participants were lower than those of nonparticipants (Table S3). The adult and other‐type circles of Willis were pooled in a single category. Thus, ΔBA diameter was compared between the 2 groups; patients with the adult or other‐type circle of Willis and those with the fetal‐type circle of Willis. During the follow‐up period, BA diameter in patients with the adult/other‐type circles of Willis increased, whereas there was almost no change in patients with the fetal‐type circle of Willis (mean±SD, 0.41±0.46 mm versus 0.04±0.31 mm; *P*=0.013; Figure 2A). Moreover, an inverse correlation was observed between the initial BA diameter and the ΔBA/y in patients with adult/other‐type circles of Willis (*P*=0.002, *r*=−0.25; Figure 3). Variables relevant to the ΔBA/y are shown in Table 2. Traditional vascular risk factors were not associated with ΔBA/y. In the multivariate analysis including sex, history of CVD, estimated glomerular filtration rate, mean maximum intima‐media thickness, and MRI parameters (ie, initial BA diameter or the type of the posterior circle of Willis), men (β=0.26, *P*=0.004), an initial BA diameter (β=−0.29, *P*=0.002), and fetal‐type circle of Willis (β=−0.24, *P*=0.004) were found to be independently associated with ΔBA/y (Table 3).

**Table 1:** Baseline and Follow‐Up Characteristics of Patients With MRI Follow‐Up (n=164)

Characteristics	Baseline	Follow‐Up
Age, y	65±7	74±7
Male sex	97 (60)	…
Risk factors		
Hypertension	123 (80)	138 (84)
Dyslipidemia	95 (62)	103 (64)
Diabetes mellitus	42 (27)	64 (40)
History of cardiovascular disease	68 (41)	92 (56)
Current smoking	26 (16)	7 (4)
Systolic blood pressure, mm Hg	138±19	137±17
Diastolic blood pressure, mm Hg	81±12	76±11
Body mass index, kg/m^2^	23.3±2.7	23.2±3.3
eGFR, mL/min per 1.73 m^2^	69±20	63±17
LDL cholesterol, mg/dL	127±35	103±28
HDL cholesterol, mg/dL	56±18	56±18
HbA1c, %	6.0±1.0	6.3±1.0
Fasting blood glucose, mg/dL	110±27	115±34
Mean maximum IMT, mm	1.07±0.39	1.33±0.45
BA diameter, mm	2.7±0.8	3.1±0.8
Fetal type (n=10 in each group)	2.0±0.5	2.0±0.3
Adult type (n=120 in each group)	2.9±0.7	3.4±0.7
Other type (n=34 in each group)	2.1±0.6	2.5±0.6
PVH and DWMH score, median (IQR)	5 (8)	8 (11)
PVH score, median (IQR)	3 (3)	3 (2)
DWMH score, median (IQR)	2 (7)	6 (8)
Lacunar infarction	39 (24)	58 (38)

Data are presented as mean±SD or number (percentage) for continuous and categorical variables, respectively. BA indicates basilar artery; DWMH, deep white matter hyperintensity; eGFR, estimated glomerular filtration rate; HbA1c, glycosylated hemoglobin A1c; HDL, high‐density lipoprotein; IMT, intima‐media thickness; IQR, interquartile range; LDL, low‐density lipoprotein; MRI, magnetic resonance imaging; PVH, periventricular hyperintensity.

**Table 2:** Variables Relevant to ΔBA (Univariate Analysis, n=164)

Variables	ΔBA Diameter, mm		*P* Values
	Mean±SD	*R*	
Age	…	−0.06	0.465
Sex, male/female	0.05±0.06/0.03±0.04	…	0.110
Risk factors			
Hypertension, yes/no^a^	0.04±0.05/0.03±0.04	…	0.288
Dyslipidemia, yes/no^a^	0.04±0.05/0.04±0.05	…	0.871
Diabetes mellitus, yes/no^a^	0.04±0.05/0.04±0.05	…	0.968
History of cardiovascular disease	0.04±0.05/0.05±0.05	…	0.063
Average body mass index, kg/m^2^	…	−0.01	0.873
Average eGFR, mL/min per 1.73 m^2^ a	…	0.19	0.014
Type of circle of Willis			
Fetal	0.001±0.03	…	…
Adult	0.05±0.05	…	…
Other	0.05±0.04	…	…
PVH score+DWMH score	…	−0.001	0.991
PVH score	…	0.05	0.581
DWMH score	…	−0.01	0.867
Lacunar infarction, yes/no	0.04±0.05/0.05±0.05	…	0.246
Mean maximum IMT	…	−0.11	0.165

Data are presented as mean±SD for continuous and categorical variables. ΔBA indicates change in basilar artery; eGFR, estimated glomerular filtration rate; DWMH, deep white matter hyperintensity; IMT, intima‐media thickness; PVH, periventricular hyperintensity.

a If a patient had treatment for more than half of the follow‐up period, the patient had hypertension/dyslipidemia/diabetes mellitus during the observational period.

**Table 3:** Multivariate Analysis Among ΔBA and Other Variables (n=164)

Variables	Coefficient (95% CI)	*P* Value
Sex (male)	0.26 (0.09 to 0.44)	0.004
History of CVD	−0.07 (−0.24 to 0.09)	0.356
Type of circle of Willis (fetal type)	−0.24 (−0.40 to −0.08)	0.004
Initial BA diameter, mm	−0.29 (−0.46 to −0.11)	0.002
eGFR, mL/min per 1.73 m^2^	0.02 (−0.13 to 0.17)	0.812
Mean maximum IMT	−0.14 (−0.31 to 0.03)	0.098

ΔBA indicates change in basilar artery; CVD, cardiovascular disease; eGFR, estimated glomerular filtration rate; IMT, intima‐media thickness.

### Comparison Between Patients With and Without CVEs in 164 Patients

Forty‐seven patients (29%) developed new CVEs during the follow‐up period. Of the 47 patients, 30 experienced cerebrovascular events (transient ischemic attack in 7, lacunar infarction in 5, large‐artery atherosclerosis in 7, cardioembolism in 4, and other or unknown infarction in 7), 15 experienced coronary events (acute myocardial infarction in 2 and revascularization therapy for ischemic heart disease in 13), and 2 experienced peripheral arterial events. Lacunar infarctions were more likely to be observed in patients with CVEs (51% versus 33%; *P*=0.043; Table 4). The ΔBA/y in patients with CVEs was smaller than that in patients without CVEs, excluding the patients with the fetal‐type circle of Willis (mean±SD, 0.02±0.10 mm versus 0.05±0.05 mm; *P*=0.010; Table 4, Figure 2B).

**Table 4:** Difference Between Patients With or Without CVEs (n=164)

Variables	With CVEs (n=47)	Without CVEs (n=117)	*P* Values
Age, y	66±7	64±7	0.219
Sex (male)	31 (66)	66 (56)	0.475
Risk factors			
Hypertension^a^	37 (80)	91 (79)	1.000
Diabetes mellitus^a^	15 (31)	33 (28)	0.705
Dyslipidemia^a^	19 (40)	59 (51)	0.229
History of CVD	23 (47)	45 (39)	0.471
PVH score+DWMH score, median (IQR)	6 (11)	4 (8)	0.525
PVH score	3 (2.75)	2 (2)	0.107
DWMH score	4 (8)	2 (7)	0.083
Lacunar infarctions	22 (51)	36 (33)	0.043
Type of ∼ of circle of Willis (fetal type)	1 (2)	9 (8)	0.284
Average eGFR, mL/min per 1.73 m^2^ a	67±20	70±20	0.839
Initial BA diameter, mm	2.9±0.9	2.6±0.7	0.060
Annual change in BA diameter, mm^b^	0.02±0.10	0.05±0.05	0.010

Data are presented as mean±SD or number (percentage) for continuous and categorical variables, respectively. BA indicates basilar artery; CVD, cardiovascular disease; CVE, cardiovascular event; DWMH, deep white matter hyperintensity; eGFR, estimated glomerular filtration rate; IQR, interquartile range; PVH, periventricular hyperintensity.

a If a patient had treatment for more than half of the follow‐up period, the patient had hypertension/dyslipidemia/diabetes mellitus during the observational period.

b Patients with the fetal variant of the circle of Willis were excluded from the analysis.

## Discussion

In this study, we found that the BA diameter gradually increased over a median period of 9 years in patients with the adult or other‐type circle of Willis. Male sex, a smaller initial BA diameter, and the type of the circle of Willis (excluding the fetal type) independently increased ΔBA. We also found that a smaller ΔBA/y was significantly associated with a high risk of CVEs.

This is the first study to demonstrate longitudinal ΔBA. Our study shows that the progression of BA dilatation was associated with men but inversely associated with initial BA diameter and fetal‐type circle of Willis. Our findings are in line with several cross‐sectional studies that also discovered that the following factors were relevant to BA diameter (ie, male sex^3^, ^5^, ^10^ and type of the circle of Willis^10^, ^18^). An association between BA diameter and severity of WMH was also observed in some studies,^7^, ^10^, ^19^ including our previous report, whereas an association between ΔBA and severity of WMH was not observed in this study. The patients we selected for the analysis of longitudinal ΔBA were younger and had lower PVH and DWMH scores (Table S3), which may be reasons for this result. However, this cannot be definitely concluded because this is the first study on the topic and the sample size analyzed was small. WMH is one of the neuroimaging features of small‐vessel disease on MRI.^20^ BA dolichoectasia and IADE have been shown to be involved with small‐vessel disease in several studies.^5^, ^6^, ^7^, ^8^, ^19^, ^21^ The underlying pathogenesis is still unclear, although several relevant factors have been proposed: increased arterial stiffness, vascular resistance,^10^, ^22^, ^23^ and the contribution of matrix metalloproteinase.^6^ In addition, the BA diameter was significantly smaller in the fetal‐type than in the adult or other‐type circle of Willis, which was the same as in previous reports.^10^, ^18^ BA dilatation is influenced by the type of circle of Willis; blood flow or wall shear stress may affect the BA diameter. Our result on the longitudinal ΔBA strengthens this finding.

In the current study, we found that the larger the initial BA diameter, there was a smaller ΔBA. This may suggest the hypothesized ceiling effect in which BA progression reaches a certain threshold at advanced ages. This is in line with an autopsy study finding in which the outer diameter in cerebral arteries remains relatively constant with age while elastin loses its functionality over time.^24^ However, although the initial BA diameter had an association with the incidence of CVEs, we found an unusual effect of ΔBA on the incidence of CVEs. A larger ΔBA was associated with the reduced risk of CVEs. Positive remodeling may be a cause of BA dilatation within the normal range.

After the addition of the initial BA diameter in the model (Figure 1), however, the association between ΔBA and CVEs disappeared. Although the diameter changed during the follow‐up, the change was small despite the long‐time duration, which is why the effect of ΔBA alone on CVEs appears to be small. Moreover, initial BA diameter and ΔBA had a significant correlation. The initial BA diameter was an important predictor of CVE risk, independent of age. Together, these results link the progression of BA diameter with the inverse rates of CVEs and provide novel insights into the clinical impact of IADE. None of the previous articles analyzed longitudinal ΔBA in relation to CVEs; thus, comparisons are limited. Nonetheless, it may be a worthwhile metric to assess ΔBA for vascular burden in patients with vascular risk factors. Further consideration is necessary to confirm our results.

In this study, we found an association between ΔBA and CVEs, particularly in coronary heart events. Swartz^25^ also reported that the BA and coronary arteries might dilate and remodel in similar ways and that each reflected the risk of the other.^2^ Moreover, the diameter of the BA and that of the right coronary artery were positively correlated in a large consecutive autopsy series.^26^ In contrast, absence of a relationship between ΔBA and cerebrovascular events might have occurred because stroke is a multifactorial disease and by the lack of statistical power to detect an association.

The strengths of this study included its longitudinal observation of participants, systematic clinical information during the follow‐up period, and the comprehensive MRI evaluation by trained raters using validated scales for small‐vessel disease. On the other hand, we had some potential limitations in the study. First, all participants had ≥1 vascular risk factor. Therefore, the results might have been different if healthy people were assessed. Second, as for the analysis of 164 patients, the subjects only included patients who could continue to visit the hospital and excluded those who died or had severe disabilities that prevented hospital visits. This sampling bias and small sample size might have affected our results. Third, we defined the BA diameter as the minor axis of the BA diameter on the axial MRI images because the BA sometimes extends obliquely and is shaped like an ellipse in a cross‐sectional view.^10^ To verify the validity of the BA diameter measurement on T2‐weighted images, we compared the diameter of BA on the slice of T2‐weighted images with time‐of‐flight MR angiograms based on 30 random subjects. Intraobserver variability of the methods was determined by intraclass correlation coefficients and Bland‐Altman plots; intraclass correlation coefficient=0.95, and Bland‐Altman plots are shown in Figure S2. In this context, MRI measurements were made using clinical scans from T2 black voids, which may be as valid as time‐of‐flight MR angiograms to measure the actual luminal diameter. Because there is no consensus on the optimal method of identifying and measuring the BA,^1^ one cannot directly compare our findings with those from other studies that used different definitions. Fourth, we were unable to assess plaque in the BA. Intracranial artery remodeling in response to plaque formation, and the BA, has a greater capacity for positive remodeling.^27^ The correlation between IADE and plaque in the BA had been reported.^6^ We also need to investigate intravascular lumen by using black blood MRI, for example. Finally, we could not investigate pathological data.

In conclusion, during the follow‐up period, the BA diameter in patients with the adult/other‐type circles of Willis gradually increased. The smaller ΔBA had an association with the high risk of CVEs. ΔBA could be a potential predictor of CVEs; however, prospective studies of larger sample sizes, including pathological assessments, are necessary in the future to clarify which has a more important relationship with CVEs.

## Sources of Funding

Part of this work was supported by the following grants: Grant‐in‐Aid for Scientific Research (C) (JP 15K19227, JP 17K09084) (Miwa) from “Ministry of Education, Culture, Sports, Science, and Technology.”

## Disclosures

None.

## Supporting information


**Table S1.** Characteristics of Patients (n=493)
**Table S2.** The Details of the Cardiovascular Events During the Follow‐Up Period (n=493)
**Table S3.** Difference Between Patients With and Without Follow‐Up MRI
**Figure S1.** Flow chart of patient enrollment in this study.
**Figure S2.** Bland‐Altman plots of measured basilar artery (BA) diameters based on T2‐weighted images and time‐of‐flight (TOF) magnetic resonance angiography (MRA).
