# Supplementary material for: A 9‐Year Longitudinal Study of Basilar Artery Diameter
Source: J Am Heart Assoc. 2019 Feb 23;8(5):e011154. doi: 10.1161/JAHA.118.011154 (PMC6474931; doi:10.1161/JAHA.118.011154)

SUPPLEMENTAL MATERIAL

Table S1. Characteristics of Patients (n = 493) .

Age, y	68 ± 7
Male sex	58%
Risk factors	
Hypertension	80%
Dyslipidemia	73%
Diabetes mellitus	24%
History of cardiovascular disease	52%
Current smoking	19%
Body mass index, kg/m^2	23.0 ± 2.8
eGFR, ml/min per 1.73 m^2	68 ± 18
LDL-cholesterol, mg/dl	128 ± 34
HDL-cholesterol, mg/dl	62 ± 17
HbA1c, %	6.0 ± 1.0
Fasting blood glucose, mg/dl	108 ± 30
Mean max IMT, mm	1.08 ± 0.37
BA diameter, mm	2.7 ± 0.7
Fetal type, n	20, 1.7 ± 0.4
Adult type, n	285, 3.0 ± 0.6
Other type, n	188, 2.5 ± 0.7
PVH, median (IQR)	3 (4)
DWMH, median (IQR)	4 (9)
Lacunar infarction, %	28%

Data are presented as mean (SD) or n (%) for continuous and categorical variables, respectively.

eGFR indicates estimated glomerular filtration rate; LDL, low-density lipoprotein cholesterol; HDL, highdensity lipoprotein cholesterol; HbA1c, glycosylated hemoglobin A1c; IMT, intima-media thickness; BA, basilar artery; PVH, periventricular hyperintensities; IQR, interquartile range; and DWMH, deep white matter hyperintensities.

Table S2. The details of the cardiovascular events during the follow-up period (n = 493).

events	n = 105
Cerebrovascular events	n = 58
TIA, n	7
Lacunar infarction, n	15
Large-artery atherosclerosis, n	14
Cardioembolism, n	9
Other or unknown, n	10
Cerebral hemorrhage, n	2
subarachnoid hemorrhage, n	1
Coronary events	n = 41
Acute myocardial infarction, n	10
revascularization therapy for ischemic heart disease, n	31
Peripheral arterial events	n = 6

TIA indicates transient ischemic attack.

Table S3. Difference between patients with and without follow-up MRI.

	Patients with MRI* (n = 164)	Patients without MRI* (n = 329)	P value
Age, y	67 ± 7	70 ± 8	<0.0001
Male sex	61%	56%	0.381
Vascular risk factors			
Hypertension	82%	78%	0.340
Dyslipidemia	68%	76%	0.080
Diabetes mellitus	25%	24%	0.822
History of cardiovascular disease	36%	33%	0.542
Current smoking	24%	18%	0.141
Body mass index, kg/m^2	23.5 ± 2.9	22.8 ± 2.7	0.005
eGFR, ml/min per 1.73 m^2	69 ± 17	67 ± 18	0.077
LDL-cholesterol, mg/dl	126 ± 35	128 ± 34	0.695
HDL-cholesterol, mg/dl	56 ± 16	57 ± 18	0.882
HbA1c, %	6.0 ± 1.0	6.0 ± 0.9	0.752
Fasting blood glucose, mg/dl	108 ± 29	107 ± 30	0.534
Mean max IMT, mm	1.05 ± 0.36	1.10 ± 0.37	0.068
BA diameter, mm	2.7 ± 0.8	2.7 ± 0.7	0.249
Fetal type, %	5%	4%	0.637
PVH, median (IQR)	3 (3)	3 (3)	0.002
DWMH, median (IQR)	2 (7)	4 (9)	0.007
Lacunar infarction	26%	28%	0.666
Cardiovascular events during follow-up period	19%	18%	0.804

*MRI; MRI was undergone at least 7.5 years between baseline and last follow-up MRI

Data are presented as mean (SD) or n (%) for continuous and categorical variables, respectively. MRI indicates magnetic resonance imaging; eGFR, estimated glomerular filtration rate; LDL, low-density lipoprotein cholesterol; HDL, high-density lipoprotein cholesterol; HbA1c, glycosylated hemoglobin A1c; IMT, intima-media thickness; BA, basilar artery; PVH, periventricular hyperintensities; and DWMH, deep white matter hyperintensities.

Figure S1. Flow chart of patient enrollment in this study.

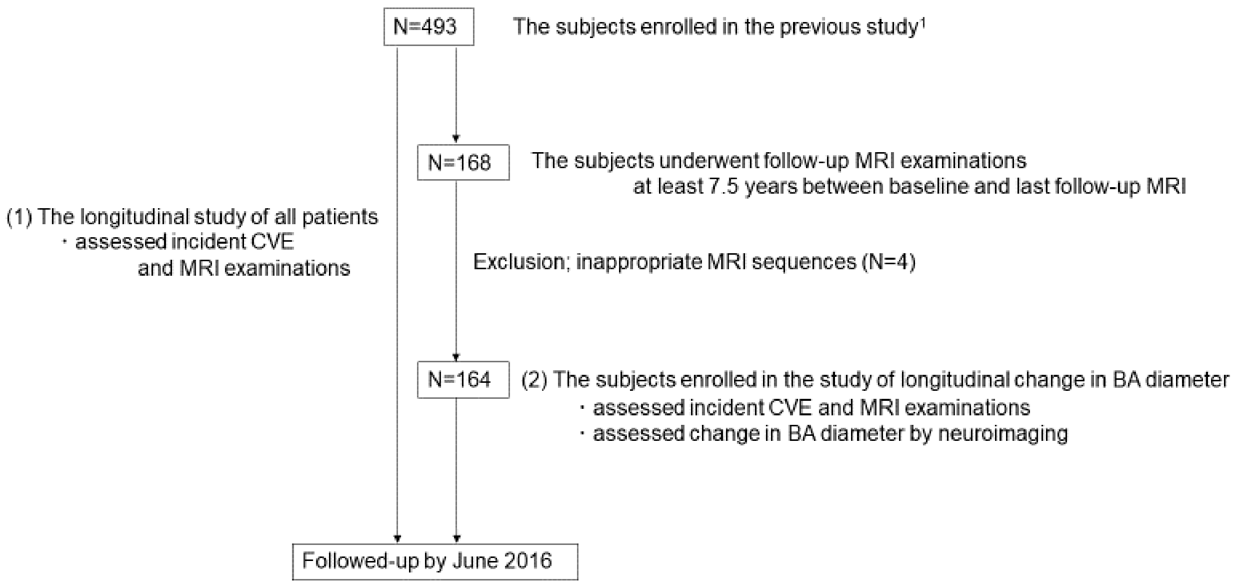

Figure S2. Bland-Altman plots of measured basilar artery (BA) diameters based on T2-weighted images and time-of-flight (TOF) magnetic resonance angiography (MRA).

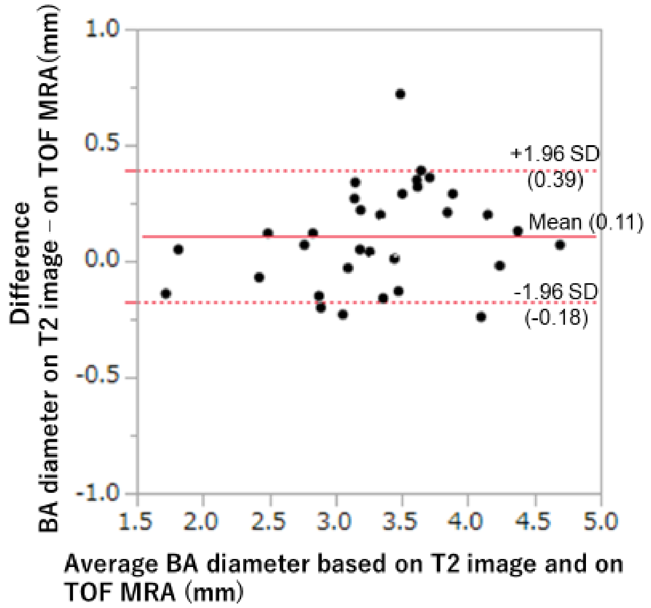

Supplement: Supplementary file 1 — Table S1. Characteristics of Patients (n=493) Table S2. The Details of the Cardiovascular Events During the Follow‐Up Period (n=493) Table S3. Difference Between Patients With and Without Follow‐Up MRI Figure S1. Flow chart of patient enrollment in this study. Figure S2. Bland‐Altman plots of measured basilar artery (BA) diameters based on T2‐weighted images and time‐of‐flight (TOF) magnetic resonance angiography (MRA). [file JAH3-8-e011154-s001.pdf]
